# A Rare Case of Pneumocystis Pneumonia in HIV Patient on Glucocorticoid

**DOI:** 10.7759/cureus.14445

**Published:** 2021-04-13

**Authors:** Usama Rehman, Khawlah Farhan, Warda Shahnawaz, Muhammad Zain Khalid, Karun Neupane

**Affiliations:** 1 Anaesthesia, Mayo Hospital, Lahore, PAK; 2 Internal Medicine, Zhejiang University, Hangzhou, CHN; 3 Medicine, Jinnah Sindh Medical University, Karachi, PAK; 4 Neurology, Liaquat National Hospital and Medical College, Karachi, PAK; 5 Internal Medicine, Manipal College of Medical Sciences, Pokhara, NPL

**Keywords:** hiv, glucocorticoid, pneumocystis jiroveci pneumonia

## Abstract

Pneumocystis pneumonia (PCP) is an opportunistic infection caused by *Pneumocystis jirovecii*. PCP due to immunosuppressive drugs is rarely reported in the literature. Herein we present a case of PCP in a 49-year-old patient who presented with progressive shortness of breath, dry cough, and low-grade fever. History revealed that he was taking prednisolone daily for his hyperactive airway disease. His temperature was 99^o^F, and he had bilateral crackles in the lungs with resonant wheezing. High-resolution computed tomography showed diffuse ground-glass haze and cystic lesions in the middle and upper zones of both lungs. He was commenced on intravenous ceftriaxone and methylprednisolone based on provisional diagnosis of interstitial pneumonia. However, his condition worsened. His human immunodeficiency virus (HIV) test was reactive, and his CD4+ count was 275 cells/mm^3^. Bronchoalveolar lavage revealed PCP by direct immunofluorescent assay. Additional serum testing revealed marked elevation of beta-D-glucan, consistent with PCP diagnosis due to glucocorticoid use. Trimethoprim-sulfamethoxazole and voriconazole were initiated, and his respiratory symptoms started improving. His respiratory condition improved on day 9, and he was discharged with follow-up.

## Introduction

Pneumocystis pneumonia (PCP) is an opportunistic infection caused by *Pneumocystis jirovecii* in immunocompromised patients, such as acquired immunodeficiency syndrome (AIDS), underlying hematologic malignancy, organ transplantation, chronic inflammatory conditions, and patients on immunosuppressive drugs [1]. It emerged at the beginning of the AIDS pandemic in 1981. A low CD4+ lymphocyte count of fewer than 200 cells/mm^3^ is a risk factor for PCP development and has become the most severe complication of the human immunodeficiency virus (HIV). However, it remains the most frequent AIDS-defining illness in the United States [2]. PCP due to immunosuppressive drugs is rarely reported in the literature [3]. Herein we present a case of PCP in HIV patient due to inappropriate use of glucocorticoids.

## Case presentation

A 49-year-old male with a past medical history of hypertension and diabetes mellitus presented with progressive shortness of breath for the last two weeks. He complained of fatigue, dry cough, and low-grade fever for the last 10 days. He had hospitalization due to a road traffic accident six months back. He was homosexual and had two male partners in the last year. His HIV antibody test was negative six months back. His history of occupational exposure or environmental hazards was unremarkable. Further history revealed that he took 20 mg prednisolone daily for his hyperactive airway disease for the last two months. He was diagnosed with community-acquired pneumonia in his local hospital and treated with intravenous ceftriaxone; however, his condition did not improve.

On examination, he had a temperature of 100^o^F, blood pressure of 110/75 mmHg, heart rate of 80 per minute, respiratory rate of 27/minute, and oxygen saturation of 95% on room air. On auscultation, he had bilateral crackles in the lungs with resonant wheezing throughout the respiration. Rest of the physical exam, including cardiovascular system and abdomen, was normal. Initial laboratory investigations revealed an elevated erythrocyte sedimentation rate (Table 1).

**Table 1:** Initial blood work-up. WBC; white blood cell, ALT; alanine aminotransferase, AST; aspartate aminotransferase, ESR; erythrocyte sedimentation rate, CRP; C-reactive protein, LDH; lactate dehydrogenase.

Parameter	Lab value	Reference range
WBC	6,171/mm^3^, neutrophil 58.2%, lymphocyte 34.3%	4,000-11,000
Platelets	301,000/mm^3^	150,000-350,000
Hemoglobin	13.1 g/dL	14-17
ALT	31 IU/L	< 35
AST	36 IU/L	< 35
Serum albumin	3.2 g/dL	4-5
Sodium	135 mmol/L	136-145
Potassium	3.7 mmol/L	3.5-5.0
Calcium	8.9 mg/dL	8.5-10.5
Creatinine	0.8 mg/dL	0.7-1.2
Blood urea nitrogen	13 mg/dL	8-20
ESR	35	< 22
CRP	11 mg/dL	< 0.2
LDH	401 IU/L	119-229

**Figure 1:**
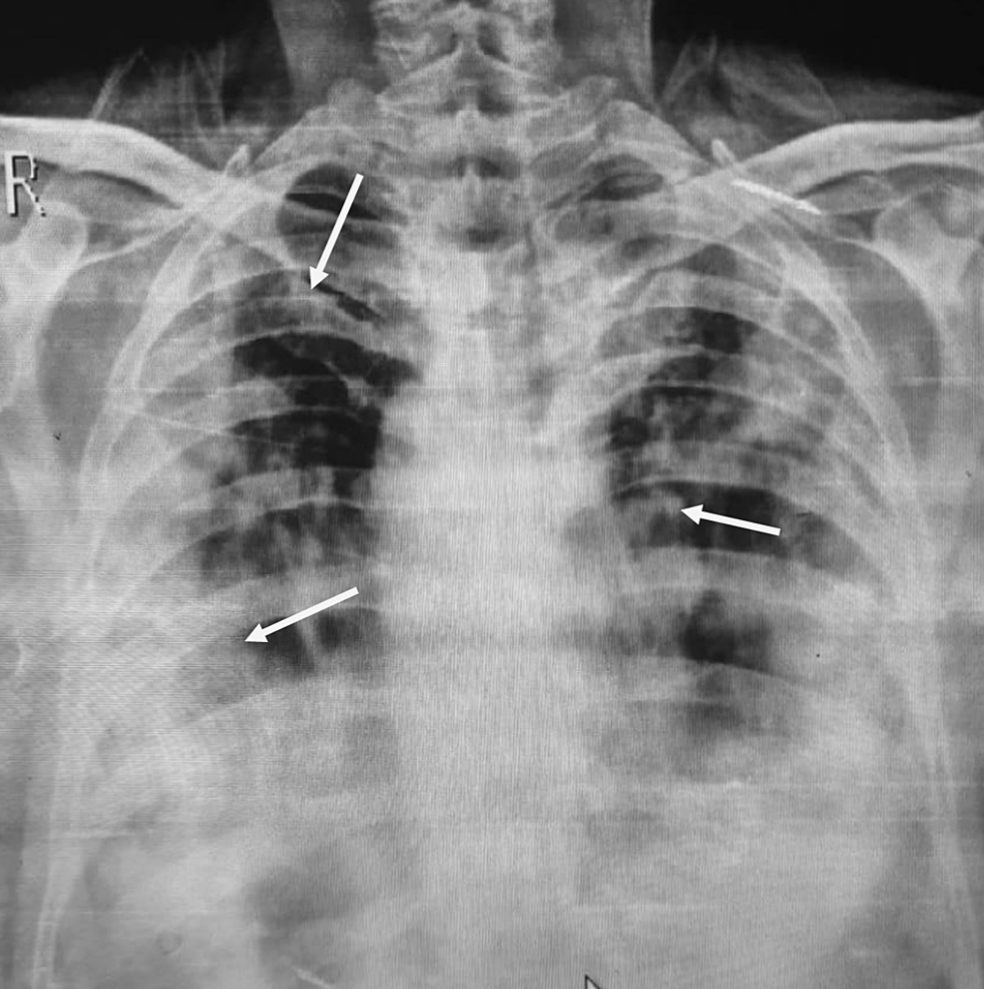
Chest x-ray showing diffuse infiltrates in both lung fields.

**Figure 2:**
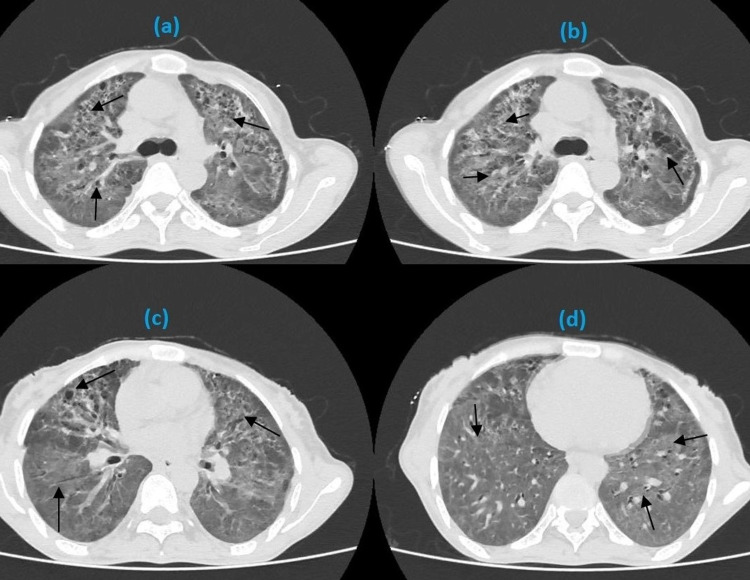
HRCT showing diffuse ground-glass haze along with bronchiectasic changes predominantly in the mid zones (a, b), and multiple cystic lesions in all zones of both lungs (a-d). HRCT; high-resolution computed tomography.

Chest x-ray revealed diffuse alveolar infiltration in both lungs (Figure 1). High-resolution computed tomography (HRCT) was performed, which showed diffuse ground-glass haze in both lung fields along with bronchiectasic changes predominantly in the mid zones. Multiple cystic lesions were also noted, mainly in the middle and upper zones of both lungs, some of which were coalescing together. Fine reticulation was also noted bilaterally (Figure 2). Initially, he was commenced on intravenous ceftriaxone and prednisolone based on the provisional diagnosis of interstitial pneumonia.

On the fourth day of admission, his condition worsened, and oxygen saturation did not improve. His blood and sputum culture were negative for any organisms. Due to the high risk of contracting HIV and the nature of the illness, he underwent HIV testing. His enzyme-linked immunosorbent assay (ELISA) was reactive, and western blot was positive. His CD4+ lymphocyte count was 275 cells/mm^3^. Bronchoscopy was performed, and bronchoalveolar lavage revealed PCP by direct immunofluorescent assay. Additional serum testing revealed marked elevation of *β*-D-glucan (BG) of 67 pg/ml, consistent with a diagnosis of PCP due to glucocorticoid use as CD4+ count was more than 200 cells/mm^3^. Trimethoprim-sulfamethoxazole (TMP-SMX) and voriconazole were initiated, and his respiratory symptoms started improving. His respiratory condition improved on day 9, and he was discharged. On follow-up after three weeks, he was doing well without recurrence of respiratory problems or evidence of immune restoration inflammatory syndrome. C-reactive protein and BG were within the normal range. His repeat HIV antibody was reactive and was commenced on antiretroviral therapy.

## Discussion

*Pneumocystis jirovecii,* previously called *Pneumocystis carinii,* affects immunocompromised patients and can be fatal in some cases [4]. Patients with an underlying disease that affect the host immune response are at high risk of contracting the disease. PCP is a fatal infection inducing inflammatory response and accumulation of fluid in the lungs leading to respiratory manifestations [5]. Although infection is rare, it spreads through the air, and in severe cases, PCP can involve other parts of the body, including the liver, bone marrow, and lymph node [1]. In immunocompromised patients, two-thirds of the cases occur in HIV patients. One-third occurs in patients who have a malignant disease, chronic inflammatory conditions, or patients on immunosuppressive drugs. The role of corticosteroids among immunosuppressive agents is prominent, and many reasons have been implicated in increased susceptibility of PCP in these patients [6,7]. One of the proposed explanations is deficient cell-mediated immunity and decreased CD4+ lymphocytes. The patient can also have associated signs and symptoms such as fatigue, headache, rash, and myalgia.

PCP usually presents with subclinical infection or obvious signs of illness. Presenting patients can have a low-grade fever, dyspnea, productive cough over several weeks. Patients typically have respiratory distress and some degree of hypoxemia while presenting in the hospital. Detailed history regarding any immunodeficiency and drug history is pertinent for appropriate clinical diagnosis of PCP [1]. Diagnosis is multifactorial and requires a multidisciplinary approach. Clinical suspicion, risk factors, laboratory investigations, imaging modalities, sputum studies, and invasive lung studies can help diagnosis. Lab workup can be non-specific, and elevated LDH and BG can be identified in the serum of the infected patients. The chest x-ray can reveal diffuse interstitial infiltrates, solitary or multiple nodules leading to cystic and cavitary lesions [8]. CT chest should be obtained for cystic lesions or ground-glass attenuation. For a definitive diagnosis of PCP, polymerase chain reaction (PCR) of respiratory specimens or fluorescein antibody staining is required [8].

Management of PCP requires early diagnosis and treatment with TMP-SMX. For mild to moderate treatment, oral treatment should be initiated, and for severe cases, intravenous TMP-SMX should be commenced [9]. Alternative therapies include atovaquone, trimethoprim, dapsone, primaquine, or clindamycin [1]. Glucocorticoids can be added to HIV-induced PCP. Prophylactic treatment is recommended for immunocompromised patients, such as those taking glucocorticoids, bone marrow suppressive therapies, or antineoplastic therapies [10]. The patient outcome depends on age, comorbidities, degree of hypoxia at presentation, other opportunistic infections, and low CD4+ count, resulting in poor outcome and mortality.

Our patient presented with non-specific clinical signs and symptoms. History of improper steroid use, lymphocytopenia, and cavitary and cystic lesion on HRCT led to the diagnosis of PCP. A strong clinical suspicion and early diagnosis and management led to the recovery of the patient.

## Conclusions

Although PCP infection is rare; however, it should be considered in the differential diagnosis of pneumonia in immunocompromised patients presenting with non-specific respiratory symptoms, lymphocytopenia. A precise and detailed history and a thorough examination is a fundamental key to diagnosis. Clinical and microbiological evaluations are indicated in high-risk patients, and BG can help diagnose PCP. Early diagnosis and management with TMP-SMZ can result in a better prognosis for the patient.
